# Predictors of *BRCA1/2* genetic testing among Black women with breast cancer: a population‐based study

**DOI:** 10.1002/cam4.1120

**Published:** 2017-06-19

**Authors:** Tarsha Jones, Anne Marie McCarthy, Younji Kim, Katrina Armstrong

**Affiliations:** ^1^ Florida Atlantic University Boca Raton Florida; ^2^ Dana Farber Cancer Institute Phyllis F. Cantor Center for Research in Nursing and Patient Care Services Boston Massachusetts; ^3^ Massachusetts General Hospital Boston Massachusetts; ^4^ Harvard Medical School Boston Massachusetts

**Keywords:** Black women, *BRCA* 1/2 testing, breast cancer, cancer prevention, genetics

## Abstract

Evidence shows that Black women diagnosed with breast cancer are substantially less likely to undergo *BRCA* testing and other multipanel genetic testing compared to White women, despite having a higher incidence of early‐age onset breast cancer and triple‐negative breast cancer (TNBC). Our study identifies predictors of *BRCA* testing among Black women treated for breast cancer and examines differences between *BRCA* testers and nontesters. We conducted an analysis of 945 Black women ages 18–64 diagnosed with localized or regional‐stage invasive breast cancer in Pennsylvania and Florida between 2007 and 2009. Logistic regression was used to identify predictors of *BRCA 1/2* testing. Few (27%) (*n* = 252) of the participants reported having *BRCA* testing. In the multivariate analysis, we found that perceived benefits of *BRCA* testing (*predisposing factor*) ([OR], 1.16; 95% CI: 1.11–1.21; *P* < 0.001), income (*enabling factor*) ([OR], 2.10; 95% CI: 1.16–3.80; p = 0.014), and *BRCA* mutation risk category (*need factor*) ([OR], 3.78; 95% CI: 2.31–6.19; *P* < 0.001) predicted *BRCA* testing. These results suggest that interventions to reduce disparities in *BRCA* testing should focus on identifying patients with high risk of mutation, increasing patient understanding of the benefits of *BRCA* testing, and removing financial and other administrative barriers to genetic testing.

## Introduction

Racial disparities in *BRCA1* and *BRCA2* genetic testing persist despite clinical availability of testing for mutations over the past 20 years 1, 2. While rates of genetic testing among women diagnosed with breast cancer appear to be increasing 3, Black women affected with breast cancer are substantially less likely to undergo *BRCA1/2* genetic testing compared to White women with the disease 2. This racial disparity is concerning as Black women have a higher incidence of early‐age onset breast cancer before age 50 (33% vs. 21.9%) 4; are twice as likely to be diagnosed with triple‐negative breast cancer (TNBC) (22 vs.11%) 5, an aggressive form of breast cancer that has been associated with a *BRCA1* gene mutation 6; and have a 42% higher mortality rate from breast cancer compared to White women 7. As recent studies have documented a high prevalence of *BRCA* and other high penetrance gene mutations among Black women with breast cancer 8, 9, 10, there is a critical need to increase uptake of genetic testing among this population to improve personalized cancer care and to reduce cancer risk.

Germline mutations in *BRCA1* and *BRCA2* tumor suppressor genes are associated with an increased risk of breast and ovarian cancers 6. *BRCA1* mutation carriers have a 55–65% risk and *BRCA2* carriers have a 45% risk of developing breast cancer by age 70 11. *BRCA1* mutation carriers have a 39% risk and *BRCA2* carriers have a 11–17% risk of developing ovarian cancer by age 70 11. Genetic testing has implications for precision prevention, as a woman who has inherited a *BRCA1* or *BRCA2* mutation can reduce her cancer risk through risk‐reducing surgeries 12, and also receive enhanced screening to promote early detection 13. There are also implications for cancer survivors long after treatment and for their at‐risk relatives who can benefit from knowing their hereditary cancer risk 14, 15.

**Figure 1:**
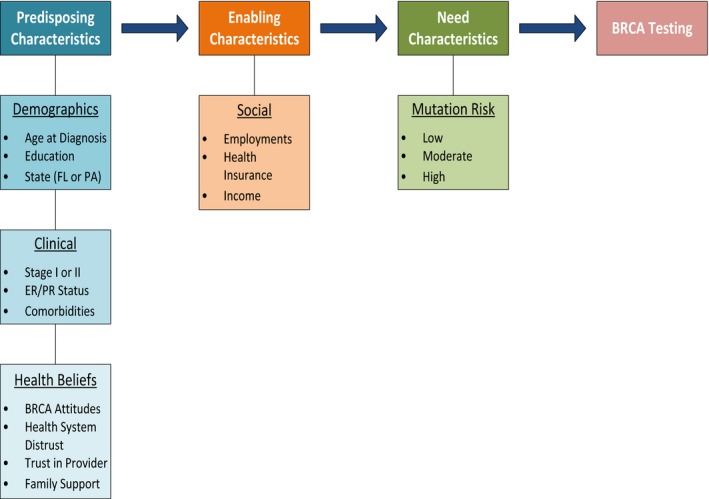
Andersen behavioral model of healthcare utilization.

The National Comprehensive Cancer Network (NCCN) guidelines recommend *BRCA1/2* testing in women diagnosed with breast cancer at age 45 and younger and those diagnosed with triple‐negative breast cancer at age 60 and younger 16. However, a recent population‐based study of over 3000 breast cancer patients found significant racial disparities in *BRCA* testing between Black and White patients that were driven by patient and healthcare‐provider‐related factors 2. The difference in use of *BRCA* testing was not eliminated after adjustment for mutation risk, sociodemographic and clinical factors, and attitudes about *BRCA* testing. The authors also found that Black women were less likely to report a recommendation from their physician to have *BRCA* testing even after adjustment for mutation risk. In this current analysis, we focus on patient‐level factors to identify predictors of *BRCA* testing among Black women with breast cancer. We also examine differences between Black women who had *BRCA* testing and those who did not. Our study is guided by the Andersen Behavioral Model of Health Care Utilization, shown in Figure 1 17. This theoretical framework posits that an individual's use of a particular healthcare service is a function of *predisposing, enabling,* and *need* factors 18.

## Methods

### Study design

We conducted an analysis of 945 Black women ages 18–64 diagnosed with localized or regional‐stage invasive breast cancer in Pennsylvania (PA) and Florida (FL) between January 1, 2007 and December 31, 2009 who were enrolled in a population‐based study. Eligibility criteria, methods of patient recruitment, and study design of this study have been previously described 2. Briefly, participants were surveyed by mail 24–36 months after cancer diagnosis with additional telephone recruitment efforts made for Black nonresponders up to 48 months after diagnosis. Women diagnosed before the age of 65 were included, to enrich the sample of women who would be appropriate candidates for genetic testing based on the NCCN Clinical Practice Guidelines 19.

**Figure 2:**
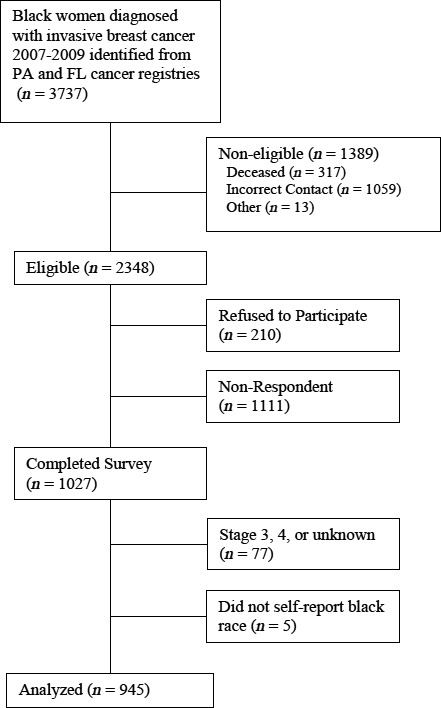
Survey responses.

The overall response rate was 61% (58% for Black women and 62% among White women) 20. Patient response rate was calculated using American Association for Public Opinion Research (AAPOR) guidelines, definition 4, which adjusts the response rate based on the estimated proportion of cases of unknown eligibility that are actually eligible 21. The responses were categorized: complete, refusal, nonrespondent, and ineligible, which included deceased respondents, those with incorrect contact information, and those with language barriers, or who reported not having been diagnosed with breast cancer. As shown in Figure 2, of the total 3737 Black patients sent a mailed survey, 1389 were ineligible, 1027 completed and returned the survey, 210 refused to participate, and 1111 were nonresponders. This resulted in the reported response rate of 58% using AAPOR definition 4. Of the 1027 respondents, participants were excluded from the current analysis if their self‐reported race was not Black (*N* = 5), or they were diagnosed with stage 3 or 4 breast cancer or unknown stage at diagnosis (*N* = 77). These exclusions resulted in the final study population of 945. This study was approved by the institutional review boards at the University of Pennsylvania, the Pennsylvania (PA) State Cancer Registry, Florida (FL) State Cancer Registry, and Massachusetts General Hospital (MGH).

### Measures

#### Sociodemographic and clinical factors

Participants completed a written survey including, but not limited to, items on patient demographics, personal and family cancer history, attitudes about *BRCA* testing, and use of genetic testing. *BRCA1/2* testing was assessed with a single item that provided the following explanation, “*BRCA* testing: this is a blood test (not a test on the tumor) that looks for genetic mutations in the *BRCA1* and *BRCA2* gene. Results can tell if a woman is at increased risk for developing ovarian cancer and a second breast cancer. Results can also help determine if relatives are at risk for these cancers.” Participants were asked if they completed *BRCA* testing. Education, employment, income, health insurance type, and comorbidities were assessed using previously established survey items. Age, stage at diagnosis, and receptor status were determined from FL and PA cancer registry data files.

#### *BRCA* mutation risk category

Risk of carrying a *BRCA1/2* mutation was categorized into mutually exclusive categories based on high, moderate, and low risk as shown in Table 1. We used age at diagnosis, family history, and Ashkenazi Jewish heritage to categorize risk based upon 2007 NCCN guidelines, which recommended genetic testing for women diagnosed with breast cancer at age 40 and younger 19.

**Table 1:** *BRCA1/2* Mutation risk categories

**High risk** – Women were categorized as high mutation risk if they met any of the five criteria below: Diagnosed ≤40 years Diagnosed ≤50 years AND: First‐ or second‐degree female relative diagnosed with breast cancer ≤50 years OR First‐ or second‐degree relative with ovarian cancer First‐ or second‐degree male relative with breast cancer Two relatives with breast or ovarian cancer at any age in the same lineage Ashkenazi Jewish ancestry
**Moderate risk** – Women were categorized as moderate mutation risk if they did not meet high‐risk criteria and met either of the two criteria below: Diagnosed 41–49 years AND: A relative with breast cancer diagnosed >50 years OR No family history of cancer Diagnosed ≥50 years AND: Any family history of breast or ovarian cancer
**Low risk** – Women were categorized as low mutation risk if they did not meet criteria for high or moderate risk, that is, Diagnosed ≥50 years AND No family history of breast or ovarian cancer

#### Attitudes about *BRCA* 1/2 testing

Attitudes about *BRCA* testing were measured using a multidimensional scale that included four items about the potential benefits of testing, one item about the cost of testing as a barrier, and three items about the potential adverse effects. Participants rated their level of agreement with each item on a five‐point Likert scale. First, benefits of testing were assessed with the following four items: (1) *BRCA* 1/2 testing would help my family members manage their cancer risk; (2) My *BRCA* 1/2 test results would help me manage my cancer risk; (3) My *BRCA* 1/2 test results would help my doctor manage my cancer risk; and (4) Testing negative for a *BRCA* mutation would be reassuring about my cancer risk. Second, participants were asked the following item related to cost of testing, *BRCA* 1/2 testing is too expensive for me to afford. Third, adverse effects of testing were assessed with the following three items: (1) *BRCA* 1/2 testing would lead to problems in my family; (2) Testing positive for a *BRCA* mutation would lead to problems with my job; and (3) Testing positive for *BRCA* mutation would lead to problems with my health or life insurance. The scale was developed through extensive qualitative research with women considering genetic testing that included identification of themes and item generation. The scale was validated through an expert panel of genetic counselors and cancer genetics specialists as well as prior studies 22, 23, 24, 25, 26 showing variation across patient characteristics hypothesized to be associated with testing attitudes and through the scale's association with testing use in prior studies. The scale has been shown to have good reliability (Cronbach's Alpha = 0.75) 22, 23. Higher scores indicate more positive attitudes regarding testing. To further explore the role of attitudes toward *BRCA* testing on test utilization among Black women, we examined the subscale scores for benefits (Cronbach's Alpha = 0.91), costs (single item), and concerns (0.76) in a sensitivity analysis.

#### Healthcare system distrust

We assessed healthcare system distrust using a nine‐item scale that includes two primary domains, one that assessed values congruence (values of the healthcare system such as honesty, motives, and equity) and the other assessed technical competence of the healthcare system (Cronbach's Alpha = 0.85) 27, 28, 29, 30. Higher scores generated indicated greater distrust of the healthcare system.

#### Family support

Participants were also asked about family support using four items from a shorter version of the Perceived Social Support from Family Measure (Cronbach's Alpha = 0.73) 31, 32. Higher scores indicated greater family support.

#### Trust in provider

Trust in provider was measured using seven items from the Trust in Physician scale and measures different aspects of a trusting physician relationship from a patient's perspective (Cronbach's Alpha = 0.81) 33, 34, 35, 36. Our survey asked participants to report which doctor they are thinking about when answering the questions related to trust. Fifty‐one percent of patients referred to their oncologist, 25% referred to their surgeon, 12% referred to another type of doctor, and 12% did not specify the type of physician. Responses ranged from “strongly disagree” (one point) to “strongly agree” (five points). All items were summed; higher scores indicated more trust in provider.

### Statistical analysis

Comparisons were made between women who reported having *BRCA* testing and those who did not use two‐sample *t*‐tests for continuous variables and chi‐squared tests for categorical variables. We used descriptive statistics to characterize the study sample. Patient characteristics included age at diagnosis, stage at diagnosis (I or II) 37, ER/PR receptor status (negative, positive, unknown), education (high school or less, college, graduate school, unknown), income (<$30,000, $30,000–70,000, >$70,000, unknown), health insurance type (Medicaid, Medicare, self‐pay, employer based), state (Pennsylvania, Florida), and number of comorbidities (0, 1, 2, or more). We also compared *BRCA* testers with nontesters based on *BRCA* attitude scale (continuous), healthcare system distrust scale (continuous), family support scale (continuous), trust in healthcare provider scale (continuous), and risk group (low, moderate, high). In a multivariate analysis, we assessed the association between *predisposing* (education, comorbidities, *BRCA* attitude, trust in provider); *enabling* (insurance type and income); and *need* factors (*BRCA* mutation risk categories) and *BRCA 1/2* testing using logistic regression. Model fit was assessed using the Hosmer–Lemeshow Goodness‐of‐Fit Test. We also performed sensitivity analyses excluding women in the low mutation risk group and assessing associations of *BRCA* attitude subscales with *BRCA* testing. A two‐sided *P* < 0.05 was used as the statistical significance level. Statistical analyses were performed using STATA/IC version 14, (College Station, TX).

## Results

### Participant characteristics

A total of 945 Black women ages 18–64 diagnosed with localized or regional‐stage invasive breast cancer were included in this study. Overall, 27% (*n* = 252) of the women reported having *BRCA* testing and 73% (*n* = 693) did not have *BRCA* testing. Among women who had *BRCA* testing, 49.2% (*n* = 124) had a high *BRCA* mutation risk; 34.9% (*n* = 88) had a moderate risk; and 15.9% (*n* = 40) had a low mutation risk. Of those who reported not having *BRCA* testing, 22.2% (139) had a high *BRCA* mutation risk; 39.5% (*n* = 247) had a moderate risk; and 38.2% (*n* = 239) had a low mutation risk. Sample characteristics of *BRCA* testers and nontesters are presented in Table 2 and is organized using *predisposing, enabling*, and *need* factors based on the theoretical framework. In an unadjusted analysis, *predisposing* characteristics revealed that Black women who reported having *BRCA* testing were younger (mean age 47.2 vs. 53.2 years, *P* < 0.001); more likely to have completed graduate education (21.6 vs. 11.5, *P* < 0.001); had more positive attitudes about *BRCA* testing (28.4 vs. 25.5, *P* < 0.001); were less likely to have two or more comorbidities (17.1 vs. 20.1, *P* = 0.013); and had higher levels of trust in their healthcare providers (28.9 vs. 28.1, *P* = 0.014). Additionally, women who received testing were more likely to agree with statements of the benefits of *BRCA* testing (*P* < 0.001). There were no significant differences in agreement with *BRCA* testing being too expensive, or concerns about *BRCA* testing between women who did and did not receive testing. Among women who completed testing, *enabling* factors were being employed for wages (58.7 vs. 42.3, *P* < 0.001), having employer‐based health insurance (47.6 vs. 35.8 *P* < 0.001); and having an annual income level of >$70,000 (31.1 vs. 16.1, *P* < 0.001). After aggregating risk factors based on *need* for genetic testing, women with a high mutation risk (49.2 vs. 22.2, *P* < 0.001) were more likely to report having *BRCA* testing compared to those with lower mutation risk.

**Table 2:** Predisposing, enabling, and need characteristics of breast cancer survivors

Characteristics	BRCA testing (YES) (*n* = 252) No. %		BRCA testing (NO) (*n* = 693) No.%		*P*‐value
*Predisposing factors*					
State					
FL	149	59.1	374	53.97	0.158
PA	103	40.9	319	46.03	
Stage					
1	120	47.6	334	48.2	0.875
2	132	52.4	359	51.8	
Age at diagnosis, mean, SD	47.2 ± 8.1		53.2 ± 7.4		**<0.001**
Education					
≤High school	63	25.2	258	38.0	**<0.001**
Any college	133	53.2	343	50.5	
Graduate school	54	21.6	78	11.5	
ER/PR status					
Negative	88	34.9	193	27.9	0.105
Positive	149	59.1	458	66.1	
Unknown	15	6.0	42	6.1	
Comorbidities					
0	152	60.3	344	49.6	**0.013**
1	57	22.6	210	30.3	
2+	43	17.1	139	20.1	
BRCA attitudes scale	28.4 ± 4.1		25.5 ± 4.0		**<0.001**
BRCA benefits (family members)					
Strongly agree or agree	204	80.95	377	54.40	**<0.001**
BRCA benefits (help me manage cancer risk)					
Strongly agree or agree	191	75.79	339	48.92	**<0.001**
BRCA benefits (help my doctor manage cancer risk)					
Strongly agree or agree	203	80.56	348	50.22	**<0.001**
BRCA benefits (reassure me about cancer risk)					
Strongly agree or agree	172	68.25	346	49.93	**<0.001**
BRCA cost (too expensive for me)					
Strongly agree or agree	73	28.97	218	31.46	0.464
BRCA concerns (family problems)					
Strongly agree or agree	22	8.73	53	7.65	0.586
BRCA concerns (problems with my job)					
Strongly agree or agree	19	7.54	49	7.07	0.805
BRCA concerns (problems with my health insurance or life insurance)					
Strongly agree or agree	56	22.22	131	18.90	0.257
Healthcare system distrust scale	26.0 ± 6.0		25.6 ± 5.9		0.398
Trust in provider scale	28.9 ± 4.3		28.1 ± 4.5		**0.014**
Family support scale	16.1 ± 3.6		16.0 ± 3.5		0.598
*Enabling factors*					
Employment					
Not employed	104	41.3	400	57.7	**<0.001**
Employed for wages	148	58.7	293	42.3	
Health insurance type					
Employer based	120	47.6	248	35.8	**<0.001**
Medicaid	15	6.8	100	14.4	
Medicare	29	11.5	122	17.6	
Self‐pay	53	21.0	106	15.3	
Other/Missing	33	13.1	117	16.9	
Income					
<30K	73	30.3	302	49.8	**<0.001**
30–70K	93	38.6	207	34.1	
>70K	75	31.1	98	16.1	
*Need factors*					
Risk category					
High risk	124	49.2	139	22.2	**<0.001**
Moderate risk	88	34.9	247	39.5	
Low risk	40	15.9	239	38.2	

Boldface indicates statistical significance (*p*<.05 and *p*<0.01).

### Predictors of *BRCA* testing

In a multivariate logistic regression model of the factors that predicted *BRCA* testing (Table 3), only attitudes about *BRCA* testing, income, and *BRCA* mutation risk predicted *BRCA* testing. Women with more positive attitudes about *BRCA* testing had a significantly higher odds of testing ([OR], 1.16; 95% CI: 1.11–1.21; *P* < 0.001). In addition, women with an income of $70,000 and higher were twice as likely to have testing compared to women with a lower income ([OR], 2.10; 95% CI: 1.16–3.80; *P* = 0.014). *BRCA* mutation risk category was associated with testing and women with a high mutation risk were nearly four times likely to have testing compared to low‐risk women ([OR], 3.78; 95% CI: 2.31–6.19; *P* < 0.001). Education level, comorbidities, insurance type, and trust in provider were not significantly associated with *BRCA* testing after multivariate adjustment. Healthcare system distrust was not significantly associated with *BRCA* testing in the univariate or multivariate analyses. The Hosmer–Lemeshow goodness‐of‐fit test indicated that our model fit the data well (*P* = 0.487). We performed sensitivity analysis excluding the low mutation risk group, and the associations of predictors with *BRCA* testing were similar (data not shown). Additionally, we performed sensitivity analysis using the *BRCA* attitudes subscales of benefits, costs, and concerns (Table 3). We found that greater agreement with the benefits of *BRCA* testing was strongly associated with testing (OR = 2.28; 95% CI: 1.78–2.93; *P* < 0.001). Greater agreement that *BRCA* testing was too expensive was associated with significantly lower odds of testing (OR = 0.73; 95% CI: 0.62–0.86; *P* < 0.001). Cost of *BRCA* testing was a significant predictor of testing when entered as a continuous variable in the multivariate adjusted model, despite the fact that it did not reach statistical significance in the unadjusted analysis in Table 2, where agreement was dichotomized into only two categories. Concerns that *BRCA* testing would lead to problems with family, job, or insurance were not significantly associated with testing use. Including the *BRCA* attitudes subscales rather than the full scale did not meaningfully change the associations of other variables with *BRCA* testing (data not shown).

**Table 3:** Logistic regression model predicting *BRCA 1/2* testing adjusted for risk group, insurance type, income, education, comorbidities, BRCA attitude, and physician trust

Variables	OR	95% CI	*P*‐value
*Predisposing factors*			
Education			
≤High School	Ref		
College	1.10	0.70–1.73	0.673
Graduate School	1.44	0.77–2.67	0.252
Missing	2.31	0.30–18.03	0.425
Comorbidities			
0	Ref		
1	0.80	0.52–1.23	0.313
2+	1.06	0.62–1.81	0.843
BRCA attitudes scale	1.16	1.11–1.21	**<0.001**
BRCA attitudes scale components^a^			
Benefits	2.28	1.78–2.93	**<0.001**
Costs	0.73	0.62–0.86	**<0.001**
Concerns	0.85	0.68–1.07	0.166
Trust in provider scale	0.99	0.95–1.03	0.684
*Enabling factors*			
Insurance type			
Employer based	Ref		
Medicaid	0.50	0.23–1.09	0.081
Medicare	0.66	0.35–1.24	0.195
Self‐pay	1.36	0.84–2.19	0.210
Other/Missing	0.66	0.35–1.25	0.202
Income			
<30K	Ref		
30–70K	1.54	0.93–2.53	0.092
>70K	2.10	1.16–3.80	**0.014**
Missing	0.88	0.37–2.10	0.767
*Need factors*			
BRCA mutation risk categories			
Low risk	Ref		
Moderate risk	1.53	0.93–2.51	0.094
High risk	3.78	2.31–6.19	**<0.001**

a Logistic regression model was rerun with BRCA attitude scale components entered into the model, rather than the full scale. Odds ratios for other variables were not meaningfully changed in this model.

Boldface indicates statistical significance (*p*<.05 and *p*<0.01).

## Discussion

This study is, to the best of our knowledge, the largest population‐based study that comprehensively examined predictors of *BRCA1/2* genetic testing among Black women with breast cancer. Our study found that few (27%) Black women with breast cancer reported having *BRCA* testing. For participants who reported not having *BRCA* testing, 22% had a high mutation risk but did not receive genetic testing suggesting an unmet need. These findings indicate a critical need for healthcare providers to assess mutation probability of women with breast cancer and order genetic counseling and testing when appropriate. There is an urgent need for a greater number of Black women to receive genetic testing as they suffer disproportionately from breast cancer and experience higher rates of variants of unknown significance (VUS) compared to Caucasian populations 38.

Interestingly, after categorizing our variables using the Andersen Behavioral Model of Health Care Utilization theoretical framework and conducting a multivariate adjustment, we found that perceived attitudes about *BRCA* testing (*predisposing factor)*, income (*enabling factor*)*,* and *BRCA* mutation risk category (*need factor*) remained significant predictors of *BRCA* testing. When we examined subscales of attitudes toward *BRCA* testing, we found that perceived benefits of *BRCA* testing was positively associated with testing, while women who agreed that *BRCA* testing was too expensive had lower odds of testing. Concerns about problems raised by testing for family members, employment, and insurance were not associated with *BRCA* testing. In contrast, prior studies found that Black women expressed greater concern about the risks associated with genetic testing and genetic discrimination compared to women of other races 39, 40. One possible explanation for our finding is that Black women's awareness of *BRCA* testing and its benefits could be increasing, diminishing concerns about risks of testing. Based on our results, educating Black women about the benefits of *BRCA* testing is important and can lead to more positive attitudes and greater uptake of *BRCA* testing among this population. In one recent study among 1536 breast cancer patients, Jagsi et al. 41 found that Black and Hispanic women who had a strong desire for testing were more likely to report an unmet need for discussion about testing with a healthcare provider compared to White breast cancer patients. These findings suggest the need for healthcare providers to engage in discussions about cancer risk and the need for genetic testing with Black women who are diagnosed with breast cancer who meet the criteria for genetic testing. Existing educational and psychosocial resources offered by Facing our Risk of Cancer Empowered (*FORCE*), BrightPink Organization*,* Bring Your Brave Campaign and Know: *BRCA* Tool, which were created by the Centers for Disease Control and Prevention (CDC), can be used to increase awareness of *BRCA* and other gene tests among this population. Our study did not assess participation in genetic counseling, which has been shown to have positive psychosocial outcomes on patients 42.

Consistent with previous studies 43, 44, 45, we found that higher income was a predictor of *BRCA* testing for this population and concerns about cost were associated with lower odds of testing. Similarly, in a recent analysis of a national sample of 3628 individuals whose clinicians ordered a comprehensive *BRCA* testing, most were White, college educated, with higher incomes 44. Another recent study examined factors associated with *BRCA* testing among 440 Black breast cancer patients and found that healthcare provider referral, private health insurance, and household income greater than $35,000 were associated with genetic counseling and testing 45. Similarly, Jones et al. 43 found that among 340 young Black breast cancer survivors, income, education, and lack of access to healthcare services due to high out of pocket costs predicted *BRCA* testing. While the cost of genetic testing has substantially decreased, racial and socioeconomic disparities in the use of testing still exist 4, 46. Black women diagnosed with breast cancer with lower incomes may experience cost‐related barriers to having genetic testing. Most genetic testing companies now offer financial assistance programs to assist patients with economic barriers; healthcare providers can explore these options to assist patients in need.

*BRCA* mutation risk category, created using age at diagnosis, family history, and Ashkenazi Jewish heritage based on the NCCN 2007 clinical practice guidelines, strongly predicted *BRCA* testing in this study. As expected, Black women with a higher mutation risk based on age at diagnosis and family history were more likely to report having testing. The 2007 NCCN guidelines recommend genetic testing for women diagnosed at age 40 and younger; in our sample, approximately 30% of the Black women diagnosed with breast cancer were categorized as having a high *BRCA* mutation risk. The NCCN guidelines has been recently updated and testing is now recommended for women with a personal history of breast cancer diagnosed at age 45 and younger and individual's diagnosed with TNBC at age 60 and younger 47, potentially making more Black women eligible for testing. Despite these existing guidelines, previous studies have found that Black women diagnosed with breast cancer are less likely to receive a physician recommendation for testing compared to White breast cancer patients 2, 41, 43, 44. The role of healthcare providers in identifying appropriate individuals for testing and ensuring that genetic testing is completed to identify patients with a hereditary cancer syndrome should be emphasized.

Historically, healthcare system distrust among African Americans has contributed to lower utilization of healthcare services and lower participation in research studies 48, 49. In the context of genetic testing, Armstrong et al. 50 found that the effect of healthcare system distrust on the likelihood of testing did not differ by race (Black vs. White women) after an adjustment analysis. However, individuals who were less willing to undergo genetic testing with insurance disclosure had high values distrust and individuals less willing to undergo genetic testing from specialist had higher competence distrust. In another study, Sheppard et al. 51 found that among 100 Black women with a cancer risk, those with higher levels of medical mistrust reported lower participation in genetic counseling and testing. In our study, we found that trust in healthcare provider and healthcare system distrust were not independent predictors of *BRCA* testing in our study. One possible explanation is that while women may distrust the healthcare system, in general, they may have higher levels of trust in their particular provider, which results in women following through with testing that the provider recommends. While this current analysis focused on identifying patient‐level factors that are predictors of *BRCA* testing and did not include healthcare provider recommendation as a variable, the parent study, conducted by McCarthy et al. 2, examined racial differences in *BRCA* testing between Black and White breast cancer patients and found that having a provider recommendation for *BRCA* testing was a strong predictor of testing.

Testing for mutations in cancer predisposing genes, such as *BRCA1/2* and other high penetrance genes, has now become the standard of care for breast cancer patients with a personal and family history indicative of mutation risk 52. The numerous benefits of genetic testing, including precision medicine and precision prevention, provide compelling reasons to increase access to testing for all who will benefit 53, 54, 55. As stated in the Cancer Moonshot Blue Ribbon Panel Report, identifying individuals with a hereditary cancer syndrome is a national priority as it will allow for genetic counseling that is evidenced‐based and would promote cancer prevention and early detection leading to improved health outcomes 56. If current racial disparities in genetic testing persist among women who are diagnosed with breast cancer, the benefits and advances in genetics and precision medicine may not reach minority populations, ultimately widening the disparities gap. Additionally, rates of variants of unknown significance (VUS) are higher among Black women compared to those of European ancestry due to less testing experience 55. While most VUS are benign, some will be deleterious but still cannot be used for clinical decision making 52. The identification of Black women with a breast cancer susceptibility gene will advance our understanding of the genetic influences of breast cancer in this population, thus leading to greater cancer prevention efforts.

Evidence‐based interventions are needed to engage high‐risk Black women in having genetic testing. This study has identified relevant factors that could be the target of a theoretically based intervention. Randomized controlled trails of studies to engage Black women in cancer risk assessment and to improve uptake of genetic testing for hereditary cancer syndromes are lacking. Few promising interventions exist that have included samples of Black women with a HBOC risk or exclusively among Black women 57, 58. Mays et al. 57 evaluated the efficacy of BreastCARE intervention for women and their primary care providers. Women were randomized to BreastCARE, a tablet‐based risk assessment tool that provides tailored print out of a risk report for patients and their providers or control group that received risk assessment by telephone. The total sample (*N *= 1235) included 24% Hispanic and 22% Black women. The authors found that BreastCARE increased discussions of family cancer history, personal breast cancer risk, and genetic counseling/testing. Apple et al. 58 conducted a quality improvement project to determine the effectiveness of nurse navigators on conducting education and screening for hereditary breast and ovarian cancer syndrome and found that identifying at‐risk individuals at the time of breast biopsy impacted surgical management of patients with a hereditary risk. Joseph et al. 59 conducted a pilot randomized trial to compare two approaches for engaging 38 high‐risk low‐income women in free genetic counseling. The sample included 39% Hispanic and 13% Black women. In the first intervention arm, genetic counseling assistants contacted the participants to offer them an appointment for genetic counseling. The second intervention arm, women were mailed a printed brochure about cancer risk and the risk assessment program and were also offered a genetic counseling appointment. These existing interventions are promising in addressing the lower utilization of genetic testing among high‐risk Black women, future studies should focus on further developing and testing existing innovative interventions or programs to engage this population in having genetic testing to make informed decisions about cancer risk reduction.

The strengths of our publication are its large population‐based design and its focus on hereditary cancer risk among Black women diagnosed with breast cancer, an understudied population. Our study expands the literature as it addresses a critical barrier to progress in the field, the underutilization of genetic testing among Black women with breast cancer. Our findings present relevant factors that can be the target of a program to improve utilization of genetic testing for breast cancer risk among this population. Until the racial disparity of genetic testing is addressed, the benefits of genetic testing will not be realized for all Americans. Our study has some limitations. As breast cancer patients were surveyed several years after their cancer diagnosis and them undergoing genetic testing, we cannot establish causality between factors such as *BRCA* attitudes and genetic testing. We were also unable to obtain information from each participant's medical records on TNBC tumor subtype and did not include this as a variable, which we recognize as a limitation given the high incidence of TNBC among Black women. In addition, the results of *BRCA* testing were not ascertained at the time of the study. We focused on self‐reported use of *BRCA* testing and although the research team found that patient report of *BRCA 1*/2 testing had high positive and negative predictive values (91 and 96%, respectively) with several medical records at the Penn Medical System, we were unable to confirm testing for all participants. Although the cancer registries in FL and PA were comprised of diverse samples, we cannot be sure that our results are generalizable to other states with similar demographic patterns. Additionally, we included only Stage I and II breast cancers, and therefore our results may not be generalizable to women diagnosed with late‐stage disease.

In conclusion, Black women suffer disproportionately from breast cancer and should have genetic testing when appropriate. Our study shows that multiple factors influence uptake of *BRCA* testing among Black women diagnosed with breast cancer, such as perceived benefits of *BRCA* testing, income level, and *BRCA* mutation risk. Interventions to reduce disparities in *BRCA* testing should focus on identifying patients with high risk of mutation, increasing patient understanding of the benefits of *BRCA* testing, and removing financial and other administrative barriers to genetic testing. Women diagnosed with breast cancer, specifically those with early‐age onset breast cancer and TNBC are at‐risk for harboring a cancer predisposition gene. Therefore, it is critical that healthcare providers recognize these red flags, assess mutation probability among Black women with breast cancer, and order genetic counseling and testing when appropriate.

## Conflict of Interest

None declared.
